# A primary care approach to the management of Arthritis

**DOI:** 10.4102/safp.v62i1.5089

**Published:** 2020-02-17

**Authors:** Selvandran Rangiah, Indiran Govender, Zakariya Badat

**Affiliations:** 1 Department of Family Medicine, Faculty of Health Sciences, University of Kwazulu-Natal, Durban, South Africa; 2 Department of Family Medicine, Faculty of Health Sciences, University of Pretoria, Pretoria, South Africa; 3 Department of Family Medicine, Kalafong Hospital, Pretoria, South Africa; 4 Department of Family Medicine, Health Sciences Faculty, University of KwaZulu-Natal, Durban, South Africa

**Keywords:** osteoarthritis, rheumatoid arthritis, primary care doctor, non-steroidal anti-inflammatory drugs

## Abstract

Arthritis is a common condition seen frequently by family practitioners, and there are many types of arthritis. Management of arthritis depends largely on the specific type of arthritis that the patient suffers from. In this article, we will provide the primary care doctor with practical information for managing arthritis, focussing on the management of osteoarthritis and rheumatoid arthritis.

## Introduction

The term ‘arthritis’ refers to the swelling of a joint or joints with associated limitation of movement, heat, pain or tenderness, which is caused by inflammation or degeneration of one or more joints.^1^ It is a common cause of disability that impairs one’s physical and mental well-being, thus being a major burden on healthcare and economic resources.^2^

In the United States, the prevalence rate of self-reported arthritis is estimated to be about 59.4 million people,^3^ and the condition is a leading cause of major disability in the United Kingdom.^4^

There is a paucity of prevalence data in Africa. In South Africa, osteoarthritis is the most prevalent form of arthritis, with a prevalence rate of 55.1% in urban settings, and between 29.5% and 82.7% in adults over 65 years of age in rural settings.^5^

Arthritis is the pathological feature in over 100 different chronic diseases involving the joints and connective tissues, with the most common forms being osteoarthritis, rheumatoid arthritis (RA) and ankylosing spondylitis. Less common forms of arthritis include systemic lupus erythematosus, scleroderma, psoriatic arthritis and gout.^6^

The three major physiological categories of arthritis include *inflammatory* (rheumatoid), *degenerative* (osteoarthritis) and *crystal-induced* (gout) arthritis. The inflammatory arthritides can be further subdivided into RA (and related disorders) and connective tissue disorders.^7^

Although arthritis may be monoarticular or polyarticular, all types may be monoarticular early in the pathological course.^7^ Therefore, clinical information and imaging studies are crucial in differentiating between the different types of arthritides (Table 1).

**TABLE 1:** Classification of arthritis, clinical characteristics, lab and radiological features.^7^

Subtype	Arthritis	F:M	Age of onset (in years)	Target joints	Distribution	Radiographic features	Lab investigations
**Degen**e**rative**	OA	1:1 to 2:1	↑elderly	Lower extremity joints, PIP, DIP, first MCP joint	Asymmetrical or symmetrical	Narrow joint space; osteophytes; subchondral sclerosis	None indicated
**Autoimmune connective tissue disease**	RA	3:1	40–70	MTP, MCP, PIP, knees, hips, cervical spine	Bilateral, symmetric	Narrow joint space – symmetrical; thickened capsule; periarticular osteoporosis; marginal erosions; joint deformity	Rheumatoid factor, anti-cyclic citrullinated peptide (anti-CCP)
	SLE	9:1	30–50	MCP; PIP of the hands primarily	Bilateral, symmetric	No erosions; joint deformity; osteonecrosis	ANA, anti-ds DNA, anti-Smith antibodies, proteinuria and haematuria, serum C3/C4
**Spondyloarthropathies**	Ankylosing spondylitis	1:10	15–35	SI; spine: vertebral bodies and apophyseal articulations; hip; shoulder	Bilateral, symmetric	Erosions; periostitis; ankylosis; thin, marginal syndesmophytes	Human leukocyte antigen (HLA) B27, ESR, CRP
	Psoriatic arthritis	1:1	30–50	Predominantly upper extremity; DIP and PIP; SI; spine	Bilateral, symmetric; asymmetric in SI joints and extremities	Marginal or central erosions with periostitis; early joint space widening with eventual narrowing; non-marginal syndesmophytes; SI erosions	-
	Reiter’s syndrome	1:5	15–35	Predominantly lower extremity; MTP; calcaneus; SI; spine	Asymmetric in foot; bilateral, symmetric or asymmetric in SI joints	Similar to psoriatic in the spine and extremities; calcaneal enthesopathy	Testing for *Chlamydia, Gonorrhoea, Salmonella* and *Shigella* titers
**Crystal arthritis**	Gout	1:20	40–50	MTP of first digit; other MTP joints, DIP, midfoot, ankle, DIP joints of hand	Asymmetric; often monoarticular	Soft-tissue nodules (tophi) with calcification; para-articular erosions; intact joint space; no osteopenia	Serum uric acid, uric acid crystals in joint fluid
**Infectious arthritis**	Bacterial	Not known	↑children, elderly	Large joints – elbows, hips, knees, spine, fever	Asymmetric; often monoarticular	Effusions	Lyme disease testing, joint/blood culture
	Viral	3:1 to 4:1	↑children	Wrists, MCP, PIP, ankle, MTP	Symmetric, polyarticular, associated fever and rash	Normal	HIV, Hepatitis B surface antigen, Hepatitis C virus antibody, Parvovirus B19

*Source:* Adapted form Adams TL, Marchiori DM. Chapter 9 – Arthritides. In: DM Marchiori, editor. Clinical imaging. 3rd ed. Mosby, United States. 2014; p. 476–624. ISBN 9780323084956. https://doi.org/10.1016/B978-0-323-08495-6.00009-9

ANA, anti-nuclear antibody; CRP, C-reactive protein; DIP, distal interphalangeal joint; ESR, erythrocyte sedimentation rate; F, female; HIV, human immunodeficiency virus; M, male; MCP, metacarpophalangeal joint; MTP, metatarsophalangeal joint; OA, osteoarthritis; PIP, proximal interphalangeal joint; RA, rheumatoid arthritis; SI, sacroiliac joint; SLE, systemic lupus erythematosus; CCP, cyclic citrullinated peptide.

**FIGURE 1:**
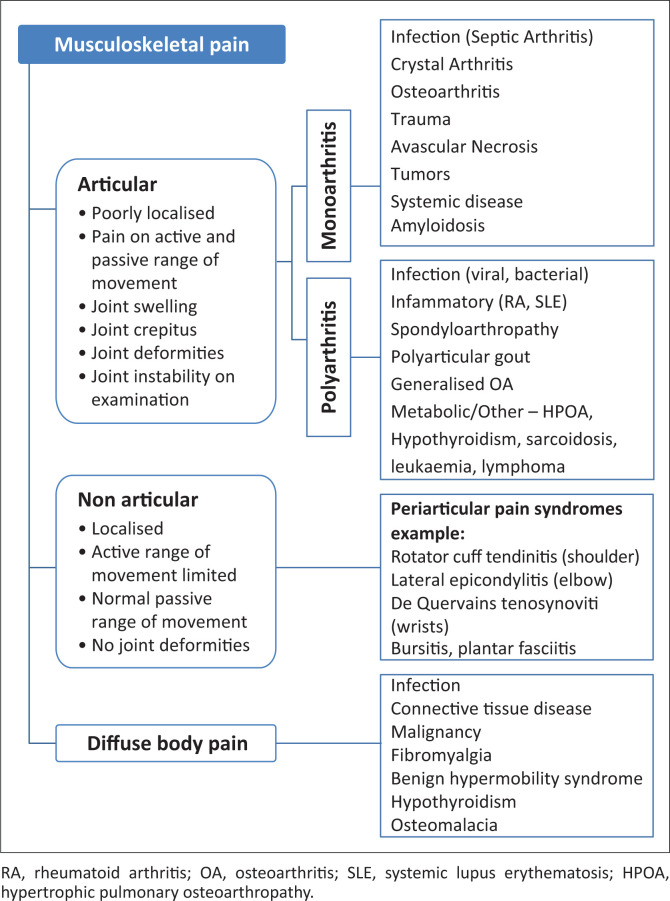
Approach to musculoskeletal pain.^8,9,10^

When approaching musculoskeletal pain, differentiating between articular and non-articular or diffuse pain narrows the differential, and this is based on history and examination (Figure 1). Articular pain in comparison to non-articular pain is more diffuse and present in both active and passive movements, while non-articular pain is usually localised to the affected structure (e.g. muscle, tendon, bursa, fascia, nerve or bone) and usually limits active rather than passive movements. In non-articular pain joint crepitus, deformity or instability is absent but muscle weakness and wasting may be present.^8,9,10^

## Osteoarthritis

Osteoarthritis is a clinical syndrome of joint pain that is accompanied by varying degrees of functional restriction, reduced quality of life and lack of psychosocial well-being.^11,12^ It is the most common form of degenerative arthritis and one of the leading causes of pain and disability worldwide.^1,11^

**FIGURE 2:**
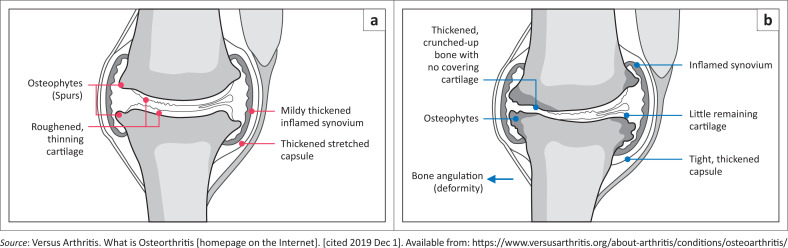
Osteoarthritis pathological findings.^14^ (a) A joint with mild Osteoarthritis and (b) a joint that has been deformed by severe Osteoarthritis.

Damage to the cartilage causes the tissues within the joint to become active, altering its structure, resulting in pain, stiffness and restricted range of movement. Osteophytes develop at the edge of the joint while the synovium thickens and fluid accumulation causes swelling (Figure 2).^13^

### Symptoms and signs

The main symptoms of osteoarthritis are pain and sometimes stiffness in the affected joints. The pain tends to be worse when the joint is moved or at the end of the day. Stiffness occurs at rest and usually improves with activity.^15^

The swelling may be hard and knobbly, which is caused by the growth of extra bone, or it may be soft, which may be caused by the thickening of the synovium and extra fluid inside the joint capsule.^16^

Crepitus is a common sign of osteoarthritis. Muscle wasting around a joint is a late feature.

### Diagnosis

The diagnosis of osteoarthritis is predominantly clinical based on a good history and physical examination. Plain radiological procedures may confirm diagnosis or rule out other medical conditions.^17^

### Management

The management of osteoarthritis encompasses both pharmacological and non-pharmacological options involving the patient in decision-making that incorporates adherence and self-management.

Key non-pharmacological interventions include arthritis education, land-based exercise programmes and weight reduction. Optional non-pharmacological interventions include heat therapy, manipulation and massage, transelectrical nerve stimulation (TENS) and water-based exercises. These can be tried according to patient preferences and stopped if found to be ineffective.^18^

The popularity of paracetamol has decreased with new studies questioning the safety profile in comparison to the minimal efficacy it produces.^19^ However, it is reasonable to trial paracetamol at a dose of up to 3 g/day and discontinue if no response is achieved.

**FIGURE 3:**
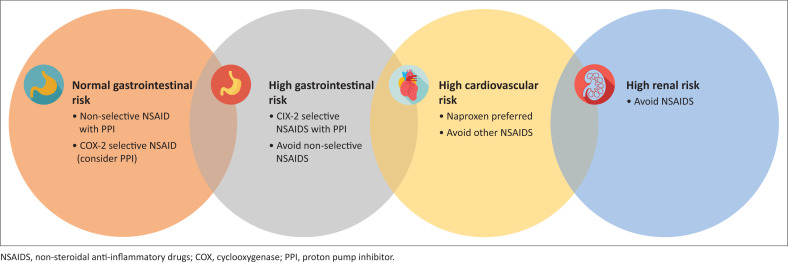
Non-steroidal anti-inflammatory drugs for knee osteoarthritis.

Topical and oral non-steroidal anti-inflammatory drugs (NSAIDS) form the backbone of pharmacological interventions for osteoarthritis. While all NSAIDS share similar efficacy, individual sensitivities may differ. In principle, the lowest dose should be used for the shortest period of time to achieve a clinical response to balance the risk of long-term use. Comorbid conditions should be factored in when prescribing NSAIDS as they are cardiotoxic, nephrotoxic and cause peptic ulceration.^20^ The conditions for co-prescribing NSAIDS with a PPI and using a selective COX-2 inhibitor are outlined in Figure 3.

Opioids provide marginal efficacy for joint pain but carry a serious risk of abuse, tramadol can be used in certain instances, while other opioids in general should be avoided. Intra-articular steroids are of significant benefit during an acute flare for short-term pain relief. However, repeated injections may result in rapid cartilage loss with no long-term benefit and hence judicious use is warranted.^21^

**FIGURE 4:**
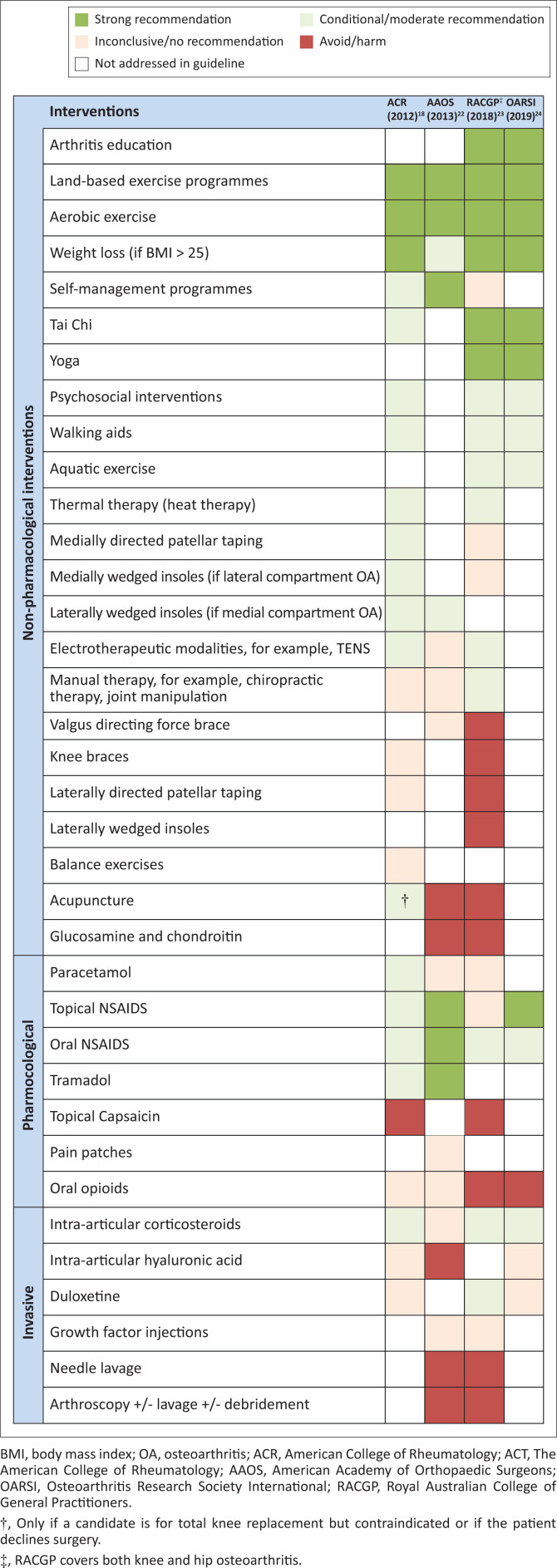
Summary of international recommendations for knee osteoarthritis management.

Figure 4 compares four international guidelines for osteoarthritis management of the knee. The American College of Rheumatology (ACR) published guidelines in 2012 for knee, hand and hip osteoarthritis.^18^ The American Academy of Orthopaedic Surgeons (AAOS) in 2013 laid out guidelines for non-surgical management of osteoarthritis of the knee.^22^ The Australian guidelines (Royal Australian College of General Practitioners [RACGP])^23^ in 2018 covered hip and knee OA and the Osteoarthritis Research Society International (OARSI) published its latest guidelines in November 2019.^24^

## Rheumatoid arthritis

Rheumatoid arthritis is a systemic autoimmune condition, which causes pain, swelling and stiffness of multiple joints of the body.^25^ Tiredness, lack of energy, weight loss, fever, sweating and dry eyes may manifest with systemic involvement. Clinical features are outlined in Table 1.

**FIGURE 5:**
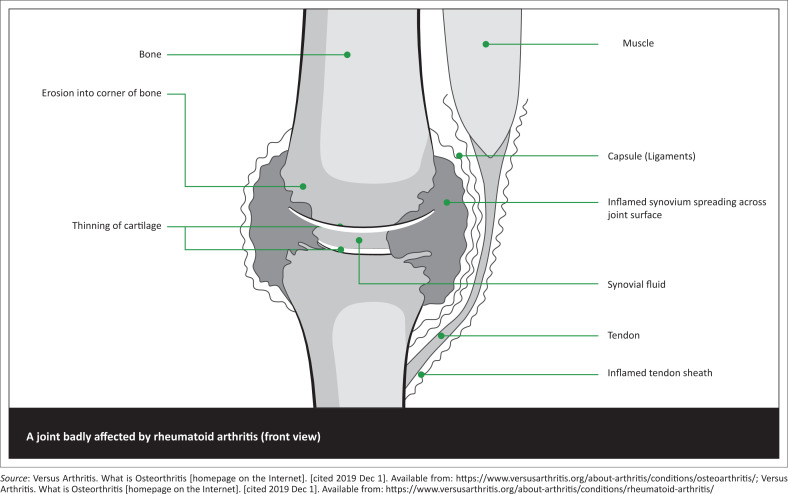
Pathological features of rheumatoid arthritis.^14^

Autoimmunity and overall systemic and articular inflammation are responsible for the destructive progression of the disease.^26^ Joint capsule instability results as the surrounding joint ligaments become weakened and stretched (Figure 5).^27^

### Diagnosis

Assessment of the patient is based on clinical history and physical examination. The pain of arthritis is the factor that causes patients to frequently seek healthcare. Characteristics such as location, quantity, intensity, nature and the course of pain can assist diagnosis. Other symptoms of arthritis include stiffness, limited motion, fatigue, weakness and swollen joints. The latter can be assessed by inspection or by direct palpation of the joint. A count of the number of swollen joints provides an indication of the amount of inflamed tissue. Use of diaries could prove a useful adjunct to traditional methods of pain assessment (e.g. visual analogue scales) and can incorporate ratings of stiffness, fatigue and mood.^27^

Early diagnosis and initiation of treatment or referral to a physician is key in the prevention of severe disability and the loss of quality of life.^27^

A joint working group of the ACR and the European League against Rheumatism (EULAR) developed updated criteria to assist in making diagnoses earlier (Table 2).^26^ Application of these criteria provides a score of 0–10, with a score of 6 or more out of 10 being indicative of the presence of definite RA.

**TABLE 2:** The 2010 American College of Rheumatology/European League against Rheumatism classification criteria for rheumatoid arthritis.^26^

Criteria	Score
**A. Joint involvement (0–5)**	
1 large joint	0
2−10 large joints	1
1−3 small joints (with or without involvement of large joints)	2
4−10 small joints (with or without involvement of large joints)	3
> 10 joints (at least 1 small joint)	5
**B. Serology (0–3)**	
Negative RF *and* negative ACPA	0
Low-positive RF *or* low-positive ACPA	2
High-positive RF *or* high-positive ACPA	3
**C. Acute-phase reactants (0–1)**	
Normal CRP *and* normal ESR	0
Abnormal CRP *or* abnormal ESR	1
**D. Duration of symptoms (0–1)**	
< 6 weeks	0
≥ 6 weeks	1

*Source:* Aletaha D, Neogi T, Silman AJ, et al. Rheumatoid arthritis classification criteria: An American College of Rheumatology/European League Against Rheumatism collaborative initiative. Arthritis Rheum. 2010;62(9):2569–2581. https://doi.org/10.1002/art.27584

Note: Add score of A to D, a score of ≥ 6/10 needed for classification of RA.

ACR, American College of Rheumatology; CCP, cyclic citrullinated peptide; ACPA, anti-cyclic citrullinated peptide antibody; CRP, C-reactive protein; ESR, erythrocyte sedimentation rate; RF, rheumatoid factor.

General laboratory tests can help determine whether arthritis and its treatment have affected major body systems. A full blood count may reveal the presence of anaemia of chronic disease, a common feature of arthritis. Neutropenia is often associated with severe RA. Liver function tests and viral serology are essential prior to commencement of treatment.^26^

In RA, 80% of patients will test positive for rheumatoid factor. Positive rheumatoid factor and anti-citrullinated peptide antibody tests are associated with increased disease severity, the development of erosions, extra-articular manifestation and greater disability. Levels of acute phase reactants (e.g. C-reactive protein and erythrocyte sedimentation rate) are used to monitor disease activity.^23,25^ Radiography, or X-rays, is helpful both diagnostically and in monitoring the disease progression.

### Management of rheumatoid arthritis

The management of RA has changed dramatically over the past 30 years.^28^ The fundamental principles revolve around disease control within 3–6 months, with the goal being remission or a low disease activity state.^28^

The 2016 update of the EULAR recommendations is based on recent evidence in the area of RA management.^29^ The principles explain the importance of early referral to a rheumatologist (or family physician or internal medicine consultant in settings with restricted access, such as the South African district health system) so that targeted therapy can be initiated based on the principles of monitoring disease activity and shared decision-making.

The recommended target is to reach a state of sustained remission or low disease activity using standardised scoring systems like the disease activity 28 joint score (DAS28). Methotrexate (MTX) is the initial drug of choice if not contraindicated. It is a conventional synthetic disease-modifying antirheumatic drug (csDMARD). A trial period of 3 to 6 months is the goal before treatment is adjusted according to the degree of improvement.

A second-line drug like another csDMARD (e.g. leflunomide or sulphasalazine) may be added in the presence of poor prognostic factors. Biological drugs (e.g. etanercept) may be added to csDMARDS until remission before the former is weaned off and then the latter.

Biologicals should be co-prescribed with a csDMARD in most cases. It is not advisable to combine two biologicals. Once remission is obtained, first wean off the biological and then the csDMARD.

A multidisciplinary approach through networking with allied health disciplines like physiotherapy, occupational therapy, podiatry and dietetics is essential to reach goal-orientated targets.^8^ Patient education directed at strengthening social and emotional support to enhance self-management of pain and disability as well as adherence to health recommendations is essential.^25^

## Summary

The basic management for some of the other common arthritis is detailed in Table 3.

**TABLE 3:** Management of other arthritides.

Arthritis	Therapeutic Options
**SLE**	Hydroxychloroquine, glucocorticoids; if they fail, MTX or AZA; biological – Belimumab^30^
**Ankylosing spondylitis**	NSAIDS
	TNF Inhibitors, for example, infliximab, etanercept, adalimumab
	IL17 inhibitors – secukinumab, ixekizumab^31^
**Psoriatic arthritis**	TNF inhibitor biologics
	Oral small molecules – MTX, SSZ, CYC
	IL12/23i – ustekinumab
	IL17 inhibitors
	Smoking cessation^32^
**Reiter’s syndrome**	Acute – NSAIDS
	Chronic stage – DMARDS (SSZ, MTX)
	Biologics – etanercept, adalimumab
**Gout**	Acute attack – NSAIDS, systemic steroids if cannot tolerate, or no response use colchicine
	Recurrent attacks (2 or more/year), tophi, urate arthropathy or kidney failure – urate-lowering therapy with allopurinol (caution – nephrotoxic) use probenecid^33^
**Bacterial**	Drainage of infected fluid
	Antibiotics
	Joint immobilisation
**Viral**	Treat the cause
	NSAIDS

AZA, azathioprine; CYC, cyclosporine; DMARDs, disease-modifying antirheumatic drug; IL, interleukin; MTX, methotrexate; NSAIDs, non steroidal anti-inflammatory drugs; SSZ, salazopyrin; TNF, tumour necrosis factor; SLE, systemic lupus erythematosus.

## Conclusion

While arthritis is common and the differential diagnosis is broad, having a well-rounded approach and holistically managing a patient, including timely referral for the inflammatory forms of arthritis, will result in better patient outcomes, better quality of life and decreased morbidity.
